# Computational and Statistical Physics Approaches for Complex Systems and Social Phenomena

**DOI:** 10.3390/e28020217

**Published:** 2026-02-13

**Authors:** Hung T. Diep, Miron Kaufman, Sanda Kaufman

**Affiliations:** 1 Laboratoire de Physique Théorique et Modélisation CNRS UMR 8089, Université CY Cergy-Paris, 95302 Cergy-Pontoise, France; 2 Department of Physics, Cleveland State University, Cleveland, OH 44115, USA; m.kaufman@csuohio.edu; 3 Levin School of Urban Affairs, Cleveland State University, Cleveland, OH 44115, USA; s.kaufman@csuohio.edu

## 1. Introduction

Complex social and socio-technical systems have numerous interacting components, nonlinear feedback, and emergent collective behaviors. Traditional, discipline-based analyses often struggle to capture these features, particularly when interactions are heterogeneous, adaptive, and embedded in networked structures. Over the past several decades, computational and statistical physics has assisted the study of complex systems. It offers an alternative framework for addressing complexity by taking advantage of the property of complex systems in which macroscopic patterns arise from microscopic interactions. These agent-based models, borrowed from statistical physics spin models, have an advantage over other models in terms of the methods used to solve systems with microscopic interactions to obtain macroscopic phenomena such as social conflicts [1], opinion dynamics, and election outcomes [2,3,4,5]. These methods can be analytical, such as the time-dependent mean-field theory [1], or numerical, such as Monte Carlo simulations [6] and other computational methods based, for example, on the theory of probability [2,7]. The reader is referred to reviews [2,3] for a complete understanding of the recent literature and details on various methods used to treat different subjects in socio-physics.

## 2. Computational and Statistical Physics Approaches

This Special Issue, “Computational and Statistical Physics Approaches for Complex Systems and Social Phenomena (3rd Edition)”, consists of five contributions [8,9,10,11,12] which use physics-inspired methods to study a range of problems spanning political dynamics, socioeconomic distributions, network robustness, cyber–physical–social resilience, and social balance theory. Despite their different domains of interest and application, the articles share a common methodological foundation rooted in complex systems thinking, statistical modeling, and computational analysis.

The feature unifying these articles is the view that social and socio-technical phenomena are interacting systems of agents or nodes, whose collective behaviors cannot be inferred from individual components alone. Network representations are central to these studies, whether explicitly or implicitly. The emphasis is on nonlinear interactions and feedback mechanisms yielding emergent outcomes such as polarization, cascading failures, income class structure, or social balance and imbalance.

The five contributions to this Special Issue predominantly use simulation, numerical analysis, and data-driven methods, which provide empirical grounding in real-world case studies, including national election dynamics, income distributions, infrastructure systems, and benchmark networks. Statistical measures and distributions bridge micro-level modeling assumptions with macro-level observables.

Together, these articles show that statistical physics tools—such as dynamical systems, probabilistic modeling, and network analysis—can be productively transferred to the study of contemporary social and socio-technical challenges. This Special Issue aims to foster cross-fertilization between disciplines by illustrating shared methodologies applied to different areas. This shows the continued relevance of physics-based approaches to the study and understanding of complex phenomena beyond traditional physical systems.

Let us summarize the articles included in this Special Issue. Beyond the value of the present collection of socio-physics applications themselves, the respective authors have suggested some interesting directions for future research.

In [8], a statistical physics-inspired three-group model is used to simulate and anticipate polarization and potential outcomes in the 2024 U.S. elections, and explores effects of reduced polarization on results. To further explore political dynamics, the authors propose to consider multiple election cycles, instead of the single one they examined. They could also explore various aspects of social media, and the impact of asymmetric information propagation. For example, they could contribute to the study of hybrid warfare tools such as AI bots in social media used to enhance polarization by affecting citizens’ political opinions and thereby influencing election results. Various additional agent characteristics and mobility between groups could be used to relieve homophilia; and depolarization mechanisms could be evaluated for systems-level results.

In [9], a model of cascading failures in a complex cyber–physical–social system (CPSS) is applied to intelligent transport infrastructure to assess resilience and inter-node dependencies. A multi-attribute CRITIC-TOPSIS method is introduced to identify critical nodes in networks by combining multiple centrality and information metrics, tested on various real-world networks. The authors suggest the use of their model in simulated exercises to validate accuracy and their inclusion in training scenarios for specific audiences in contexts beyond the military. They could incorporate sensor, operational, or live system data into cascading failure ions, and allow agents (nodes) to change their behavior in response to failures or interventions. At the next level, human decision-making and behavioral responses during failures can be explicitly modeled. The study of failures that propagate across transport, energy, communication, and governance systems may acquire additional usefulness in the era of systems hyper-connectivity, and hybrid warfare that can race through multiple infrastructure systems.

In [10], a statistical method based on inflection points of income distribution PDFs is proposed for defining income class boundaries, validated with Brazilian national data. The next development in the direction proposed by this article could include some cross-cultural/cross-country comparisons, testing PDF-inflection-based class definitions in different economies. Class boundaries can also be tracked for shifts in response to growth, and external shocks such as policies and crises. Measures of social mobility—wealth distribution and Gini evolution—can be integrated with the class boundaries.

In [11], a multi-attribute CRITIC-TOPSIS method is introduced to identify critical nodes in networks by combining multiple centrality and information metrics, tested on various real-world networks. The authors plan to explore dynamic networks and graph neural network techniques, as well as AI. They may extend critical-node identification from static graphs to time-evolving networks, and account simultaneously for multiple interaction channels—informational, economic, social. Node-ranking methods may be integrated with the testing of robustness under targeted or random failures. Multi-criteria decision frameworks could be applied to very large real-world networks.

In [12], the application of nonlinear dynamical systems to the concept of Heider structural balance in social networks is briefly reviewed to illustrate mathematical frameworks for social stability and conflict. Empirical validation through testing predictions against real social networks is an interesting development, as is adding randomness and uncertainty to balanced evolution. Structural balance can be linked to consensus formation and polarization.

As stated above, the five articles in this Special Issue are potential starting points for several streams of future research. We need, however, to acknowledge that there are fundamental differences between social and physical systems. For example, unlike physical objects, the individuals in the networks studied have agency, and do not obey fundamental physics laws. What are the implications of this fundamental difference for socio-physics applications? For example, as seen in [1,6,8], the interaction of group A with group B can be different from the interaction of B with A (in other words, A likes B, but B does not like A). This asymmetry of interaction is not a physical law, and this issue needs further exploration.

Beyond the value of the present collection of socio-physics applications themselves, the respective authors have suggested some interesting directions for future research.

Despite the different contexts, models, and applications of specific socio-physics tools, this Special Issue’s papers share several themes. One such theme is the move from static snapshots to evolving systems. Another theme is the possible use of models as test runs for policy intervention. The empirical calibration and validation of models strengthen the credibility and usefulness of the findings. An important shared aspect of the contributions of these studies is the application of methods to real-world systems without losing interpretability. We suggest that the role of network geometry should be explored through equivalent neighbor lattice with mean field theory, Bravais lattices, and hierarchical/fractal lattices on dynamics. Monte Carlo simulations with Glauber dynamics could be used to study short-range interaction networks. For particular choices of parameters, chaotic behavior and sensitivity to initial conditions emerge. This research direction is quite important for applications, as policy makers should be able to control unpredictable chaotic situations.

As a last remark, let us mention that there is an increasing number of cross-disciplinary investigations on various social phenomena such as opinion dynamics, political polarization, and predictions of election outcomes, based on methods in social sciences (see, for example, [13,14,15,16,17,18,19]) complementary to computing methods seen in the papers published in this Special Issue.

## 3. Conclusions

To conclude, this Special Issue is especially relevant to statistical physics and computational social science researchers contending with complex systems. The interest of computing socio-physics is that it gives quantitative estimations based on a small number of relevant parameters. In particular, agent-based Monte Carlo simulations using stochastic approaches have the possibility to come closer to the reality of social and infrastructural phenomena. Complex systems promote interdisciplinarity, making them ideal objects for models in statistical physics. We have shown that these models can be analytically solved and/or numerically computed or simulated. Note that models in statistical physics are very rich, with many of them bearing aspects of social phenomena such as partial disorder and frustration [20]. They need to be interpreted in terms of social language. This is the subject of our future research.

## References

[1] Kaufman M., Diep H.T., Kaufman S. (2019). Sociophysics of intractable conflicts: Three-group dynamics. Phys. A—Stat. Mech. Its Appl..

[2] Galam S. (2012). Sociophysics: A Physicist’s Modeling of Psycho-Political Phenomena.

[3] Starnini M., Baumann F., Galla T., Garcia D., Iñiguez G., Karsai M., Lorenz J., Sznajd-Weron K. (2025). Statistical physics and beyond. arXiv.

[4] Helfmann L., Conrad N.D., Lorenz-Spreen P., Schütte C. (2023). Modelling opinion dynamics under the impact of influencer and media strategies. Sci. Rep..

[5] Bernardo C., Altafini C., Proskurnikov A., Vasca F. (2024). Bounded confidence opinion dynamics: A survey. Automatica.

[6] Diep H.T., Kaufman M., Kaufman S. (2024). Monte Carlo Study of Agent-Based Blume-Capel Model for Political Depolarization. EPJ Web Conf..

[7] Galam S. (2022). Opinion dynamics and unifying principles: A global unifying frame. Entropy.

[8] Kaufman M., Kaufman S., Diep H.T. (2025). Application of the Three-Group Model to the 2024 US Elections. Entropy.

[9] Sobb T., Turnbull B. (2025). Modelling Cascading Failure in Complex CPSS to Inform Resilient Mission Assurance: An Intelligent Transport System Case Study. Entropy.

[10] Bittencourt R., Pereira H.B.d.B., Moret M.A., Lima I.C.D.C., Galam S. (2025). A Novel Evaluation of Income Class Boundaries Using Inflection Points of Probability Density Functions: A Case Study of Brazil. Entropy.

[11] Zhao X., Zhang Y., Zhai Q., Zhang J., Qi L. (2024). A Multi-Attribute Decision-Making Approach for Critical Node Identification in Complex Networks. Entropy.

[12] Kułakowski K. (2025). Heider Balance—A Continuous Dynamics. Entropy.

[13] Klein E., Robison J. (2020). Like, post, and distrust? How social media use affects trust in government. Political Commun..

[14] Rekker R. (2021). The nature and origins of political polarization over science. Public Underst. Sci..

[15] Kubin E., von Sikorski C. (2021). The role of (social) media in political polarization: A systematic review. Ann. Int. Commun. Assoc..

[16] Maes M., Flache A. (2013). Differentiation without distancing. Explaining bi-polarization of opinions without negative influence. PLoS ONE.

[17] Dandekar P., Goel A., Lee D.T. (2013). Biased assimilation, homophily, and the dynamics of polarization. Proc. Natl. Acad. Sci. USA.

[18] Doise W. (1969). Intergroup relations and polarization of individual and collective judgments. J. Personal. Soc. Psychol..

[19] Mackie D., Cooper J. (1984). Attitude polarization: Effects of group membership. J. Personal. Soc. Psychol..

[20] Quartu R., Diep H.T. (1997). Partial order in frustrated quantum spin systems. Phys. Rev. B.

